# A Redução do Colágeno Tipo I está Associada ao Aumento da Atividade da Metaloproteinase-2 e da Expressão Proteica de Leptina no Miocárdio de Ratos Obesos

**DOI:** 10.36660/abc.20180143

**Published:** 2020-07-28

**Authors:** Danielle Cristina Tomaz da Silva-Bertani, Danielle Fernandes Vileigas, Gustavo Augusto Ferreira Mota, Sérgio Luiz Borges de Souza, Loreta Casquel De Tomasi, Dijon Henrique Salomé de Campos, Adriana Fernandes de Deus, Paula Paccielli Freire, Carlos Augusto Barnabe Alves, Carlos Roberto Padovani, Antonio Carlos Cicogna

**Affiliations:** 1 Faculdade de Medicina de Botucatu Universidade Estadual Paulista BotucatuSP Brasil Faculdade de Medicina de Botucatu - Universidade Estadual Paulista (UNESP), Botucatu, SP - Brasil; 2 Instituto de Biociências Universidade Estadual Paulista BotucatuSP Brasil Instituto de Biociências - Universidade Estadual Paulista (UNESP), Botucatu, SP - Brasil

**Keywords:** Doenças Cardiovasculares/fisiopatologia, Obesidade, Colágeno Tipo 1, Ratos, Leptina, Adiposidade, Inibidores Teciduais de Metaloproteinases, Metaloproteinase-2

## Abstract

**Fundamento:**

A obesidade é um fator de risco para complicações médicas, incluindo o sistema cardiovascular. Há informações limitadas sobre o colágeno no coração obeso. Nosso estudo anterior demonstrou uma redução dos níveis proteicos de colágeno miocárdico tipo I em ratos obesos alimentados com uma dieta com alto teor de gordura durante 34 semanas. No entanto, os mecanismos responsáveis pelos níveis baixos não estão completamente elucidados.

**Objetivo:**

O objetivo deste estudo foi testar a hipótese de que a redução do colágeno tipo I está associada ao aumento da atividade da metaloproteinase-2 (MMP-2), a qual está ligada à elevação de leptina no miocárdio de ratos obesos.

**Métodos:**

Ratos Wistar machos com 30 dias de idade foram randomizados em dois grupos: controle (dieta padrão) e obeso (dieta com alto teor de gordura), e alimentados durante 34 semanas. Foram avaliados as características gerais dos animais e os perfis metabólicos e endócrinos. Foram avaliados as expressões proteicas miocárdicas de colágeno tipo I, leptina e inibidores teciduais de metaloproteinases (TIMP), bem como a atividade da MMP-2. O teste de correlação de Pearson foi aplicado para determinar as associações entre variáveis. O nível de significância foi de 5%.

**Resultados:**

Os animais obesos apresentaram índice de adiposidade mais elevado em comparação ao controle. Foram observadas comorbidades como intolerância à glicose, hiperinsulinemia, resistência à insulina, hiperleptinemia e hipertensão nos ratos obesos. A obesidade reduziu o colágeno tipo I, TIMP-1 e TIMP-2, e aumentou a leptina e a MMP-2 no miocárdio. Houve uma correlação negativa entre o colágeno tipo I e a MMP-2 e uma correlação positiva entre a leptina e a MMP-2.

**Conclusão:**

Foi confirmada a hipótese de que a redução do colágeno tipo I está associada ao aumento da atividade da MMP-2 e da expressão de leptina no miocárdio de ratos obesos. (Arq Bras Cardiol. 2020; 115(1):61-70)

## Introdução

A obesidade é uma doença metabólica crônica caracterizada pelo acúmulo excessivo de tecido adiposo. A prevalência da obesidade tem aumentado mundialmente, representando um problema grave de saúde pública que afeta tanto países desenvolvidos quanto países em desenvolvimento.^1 , 2^

Os adipócitos são influenciados por várias substâncias, e secretam numerosos peptídeos que atuam no sistema cardiovascular de maneira direta ou indireta. Portanto, o tecido adiposo não é simplesmente um depósito de energia, mas também um órgão endócrino, parácrino e autócrino ativo com múltiplas funções, incluindo a capacidade de sintetizar e liberar mediadores, como a leptina, que participa de múltiplos processos biológicos, incluindo aqueles que ocorrem no coração.^3^

O coração é composto por miócitos, nervos, vasos e a matriz extracelular (MEC). O componente principal da MEC é o colágeno, predominantemente do tipo I e III, sendo o tipo I o mais abundante, correspondendo a aproximadamente 80% do colágeno miocárdico total.^4^ Esta proteína é produzida por fibroblastos e degradada pela família das metaloproteinases de matriz (MMP).^5^ Em uma condição estável, o colágeno contribui para a manutenção da arquitetura e função cardíaca.^6^ Vários mecanismos atuam para garantir que os componentes da degradação da matriz por MMP sejam precisamente controlados, incluindo os inibidores teciduais de metaloproteinases (TIMP).^7^ O colágeno cardíaco muda em resposta a estímulos neuro-hormonais e mecânicos,^6 , 8^ devido a síntese elevada e degradação diminuída ou vice versa.

Vários estudos têm analisado a expressão do colágeno tipo I em diferentes tecidos em modelos experimentais de obesidade.^9 - 11^ Há informações limitadas sobre o comportamento desse tipo de colágeno no coração de animais obesos. Embora Carroll et al.^12^ tenham demonstrado uma elevação em colágeno miocárdico tipo I em coelhos obesos que receberam uma dieta com alto teor de gordura durante 12 semanas, um estudo prévio do nosso grupo, Silva et al.,^13^ encontrou uma redução do colágeno miocárdico tipo I em ratos obesos que receberam uma dieta com alto teor de gorduras insaturadas durante 30 semanas.^13^ No entanto, não foram estudados os mecanismos responsáveis pela redução desse colágeno.

Um dos possíveis mecanismos envolvidos na regulação do colágeno miocárdico tipo I é o aumento do hormônio leptina.^5 , 14 - 16^ Apoiando tal hipótese, a maioria dos estudos in vitro tem demonstrado que a leptina aumenta a atividade da MMP-2,^5 , 15 , 16^ que está envolvida na degradação do colágeno tipo I. Portanto, o objetivo do presente estudo foi testar a hipótese de que a redução do colágeno miocárdico tipo I, associada com o aumento da atividade da MMP-2, está ligada à elevação de leptina em ratos obesos.

## Materiais e métodos

### Animais e protocolo experimental

Após uma semana de aclimatação, ratos Wistar machos com 30 dias de idade foram aleatoriamente designados, por sorteio, para um dos dois grupos: controle (n = 20) e obeso (n = 21). O tamanho da amostra utilizada neste estudo foi baseado na literatura e nos nossos estudos prévios.^13 , 17 - 19^ O grupo controle foi alimentado com ração de ratos padrão (RC Focus 1765, Agroceres^®^, Rio Claro, SP, Brasil) contendo 12,3% de quilocalorias de gordura, 57,9% de carboidratos e 29,8% de proteína, enquanto o grupo obeso foi alimentado com uma das quatro alternantes dietas com alto teor de gordura (RC Focus 2413, 2414, 2415 e 2416, Agroceres^®^, Rio Claro, SP, Brasil) contendo 49,2% de quilocalorias de gordura, 28,9% de carboidratos e 21,9% de proteína. As quatro dietas com alto teor de gordura tinham a mesma composição nutricional, exceto aditivos aromatizantes, a saber, queijo, bacon, chocolate ou baunilha. Cada dieta foi alternada diariamente, e os ratos mantiveram suas respectivas dietas durante 34 semanas consecutivas. A dieta com alto teor de gordura foi caloricamente rica em comparação com a dieta padrão (3,65 kcal/g vs. 2,95 kcal/g) devido à maior composição de gordura. A dieta com alto teor de gordura consistia em ácidos graxos saturados e insaturados, que forneciam 20% e 80% das calorias derivadas de gordura, respectivamente.

Os ratos foram alojados em gaiolas individuais em uma sala de ar limpo com controle ambiental a 23 (± 3)ºC com um ciclo claro/escuro de 12 horas e umidade relativa de 60% (± 5%). Todos os experimentos e procedimentos foram realizados de acordo com o Guia para o Cuidado e Uso de Animais de Laboratório, publicado pelo Conselho Nacional de Pesquisa (1996),^20^ e foram aprovados pelo Comitê de Ética da Faculdade de Medicina de Botucatu (UNESP, Botucatu, SP, Brasil, Protocolo: 861-2011).

### Características gerais dos animais e perfis metabólicos e endócrinos

As características gerais dos animais e os perfis metabólicos e endócrinos foram avaliados de acordo com os seguintes parâmetros: peso corporal, gordura corporal (GC), índice de adiposidade (IA), consumo alimentar, ingestão de calorias, eficiência alimentar, tolerância à glicose, resistência à insulina, perfil lipídico sérico e concentrações séricas de leptina e insulina. Foi aplicado um critério baseado no IA para determinar a obesidade. O IA é um método fácil e consistente utilizado por vários autores para avaliar a quantidade de GC em roedores.^21 - 23^

O consumo alimentar e peso corporal foram medidos semanalmente. Foi determinada a ingestão calórica multiplicando o valor energético de cada dieta (g × kcal) e consumo alimentar semanal. Para analisar a capacidade dos animais de converter energia alimentar consumida em peso corporal, foi calculada a eficiência alimentar, dividindo o ganho total de peso corporal (g) pela ingestão energética total (Kcal).

Foi avaliada a tolerância à glicose pelo teste oral de tolerância à glicose uma semana antes da eutanásia. Após um jejum de seis horas, foram coletadas amostras de sangue por punção da ponta da cauda na linha de base e após administração intraperitoneal de solução de glicose a 30% (Sigma-Aldrich®, St Louis, MO, EUA), equivalente a 2,0 g/kg de peso corporal. Foram analisadas as concentrações de glicose no sangue a 0 minutos (linha de base) e aos 15, 30, 60, 90 e 120 minutos de infusão de glicose, utilizando um glicosímetro portátil (Accu-chek Advantage; Roche Diagnostics Co., Indianápolis, IN, EUA). Foi avaliada a intolerância à glicose pela área sob a curva (AUC) para glicose.

Ao final do protocolo experimental, após jejum de 12 horas, os animais foram anestesiados (pentobarbital sódico 50 mg/kg, injeção intraperitoneal), decapitados e toracotomizados; os diferentes depósitos de gordura do tecido adiposo foram dissecadas e pesadas. Foi calculada a GC como a soma do peso dos depósitos de gordura individuais da seguinte maneira: GC = gordura epididimal + gordura retroperitoneal + gordura visceral. Foi calculado o IA com a seguinte fórmula: IA = (GC/peso corporal final) × 100. As amostras de sangue foram coletadas em tubos heparinizados, centrifugados a 3.000 × g por 10 minutos a 4°C e armazenados a −80°C para análise subsequente. Foram determinados triacilglicerol, colesterol total, concentrações de lipoproteína de alta (HDL) e baixa densidade (LDL) utilizando kits específicos (BIOCLIN^®^, Belo Horizonte, MG, Brasil). Os níveis hormonais de leptina e insulina foram determinados por ensaio de imunoabsorção enzimática (ELISA), utilizando kits comercialmente disponíveis (EMD Millipore Corporation, Billerica, MA, EUA).

Foi usado o modelo de avaliação da homeostase da resistência à insulina (HOMA-IR) como um índice de resistência à insulina, calculado de acordo com a fórmula: HOMA-IR = [glicose em jejum (mmol/L) × insulina em jejum (μU/mL)]/22,5.24

### Perfil cardiovascular

Foi avaliado também o perfil cardiovascular dos animais de acordo com os seguintes parâmetros: pressão arterial sistólica (PAS); morfologia do tecido cardíaco; expressão proteica miocárdica de colágeno tipo I, TIMP-1, TIMP-2 e leptina; e atividade da MMP-2.

### Pressão arterial sistólica

Ao final do experimento, uma semana antes da eutanásia, foi medida a PAS em ratos conscientes utilizando o método não invasivo de manguito de cauda com um eletro- esfigmomanômetro, Narco BioSystems^®^ (International Biomedical, Austin, TX, EUA).^25^ As pulsações arteriais foram registradas em um sistema computadorizado de aquisição de dados (Biopac Systems Inc., CA, EUA). Foi registrada a média de duas leituras para cada medida.

### Estudo morfológico

Os corações foram removidos e dissecados após a eutanásia e a toracotomia. O peso dos átrios e do ventrículos esquerdo e direito e as suas respectivas relações com o peso corporal final foram determinados com a finalidade de avaliar a presença de remodelação cardíaca (i.e., a presença ou ausência de hipertrofia).

### Níveis proteicos miocárdicos de colágeno tipo I, TIMP-1, TIMP-2 e leptina

O tecido do ventrículo esquerdo foi analisado por western blot para quantificar os níveis proteicos de colágeno tipo I, TIMP-1, TIMP-2 e leptina. Foram utilizadas seis amostras em cada grupo para garantir que todas as amostras fossem analisadas na mesma corrida de eletroforese com a finalidade de evitar variações entre géis. Resumidamente, amostras congeladas do ventrículo esquerdo foram homogeneizadas usando um dispositivo Polytron (Ika Ultra TurraxTM T25 Basic, Wilmington, EUA) em tampão de lise contendo 10 mM de Tris pH 7,4, 100 Mm de NaCl, 1 mM de EDTA, 1 Mm de EGTA, 1% Triton X-100, 10% glicerol, 0,1% dodecil de sódio sulfato de sódio (SDS), desoxicolato a 0,5% e inibidores da fosfatase e protease (Sigma-Aldrich). O homogenato foi centrifugado a 4ºC durante 20 minutos a 12.000 rpm. Foi coletado o sobrenadante e o conteúdo total de proteínas foi determinado pelo método de Bradford. As amostras (50 µg) foram submetidas a eletroforese em gel de SDS-poliacrilamida (SDS-PAGE) em géis de poliacrilamida (6% ou 10%, dependendo do peso molecular da proteína). Após eletroforese, as proteínas foram eletro-transferidas para uma membrana de nitrocelulose (BioRad Biosciences; NJ, EUA). A membrana foi subsequentemente bloqueada (5% leite em pó desnatado, 10 mmol/L de Tris-HCl pH 7,6, 150 mmol/L de NaCl, e 0,1% Tween 20) durante 2 horas à temperatura ambiente e incubada com anticorpos específicos durante a noite a 4ºC. Após isso, a membrana foi incubada durante 1,5 horas à temperatura ambiente com anticorpo secundário anti-coelho ou anti-camundongo conjugado com peroxidase (diluição 1:10.000) e subsequentemente incubada com quimioluminescência aumentada (Amersham Biosciences, NJ, USA) e detectado por autoradiografia. Foi realizada a análise de quantificação dos blots utilizando Scion Image software (Scion, baseado em NIH Image). Foram obtidos anticorpos monoclonais de camundongo para colágeno tipo I (1:10.000), TIMP-2 (1:1.000) e leptina (1:1.000) e anticorpos monoclonais de coelho para TIMP-1 (1:1.000) e β-actina (1:1.000) de Abcam (Cambridge, USA) e Cell Signaling (Danvers, USA), respectivamente. As bandas alvo foram normalizadas para a expressão da β-actina cardíaca.

### Atividade da metaloproteinase-2 miocárdica

A atividade da MMP-2 miocárdica foi determinada conforme descrito por Tyagi et al.^26^ Foram usadas seis amostras em cada grupo para garantir que todas as amostras fossem analisadas na mesma corrida de eletroforese para evitar variações entre géis. Resumidamente, os tecidos do ventrículo esquerdo foram homogeneizados em um tampão contendo: Tris 50 mM, pH 7,4, NaCl 0,2 M, Triton-X 0,1% e CaCl_2_ 10 mM. O homogenato foi centrifugado a 4°C durante 20 minutos a 12.000 rpm. O sobrenadante foi coletado, e o conteúdo total de proteínas foi determinado pelo método de Bradford (Bradford 1976). As amostras foram diluídas em um tampão de aplicação contendo: 0,5 M Tris, pH 6,8, 100% glicerol e 0.05% azul de bromofenol. As amostras foram carregadas em poços de SDS-poliacrilamida a 8% contendo gelatina a 1%. Foi realizada a eletroforese em um dispositivo Bio-Rad a 80 V durante duas horas. O gel foi removido e lavado duas vezes com Triton-X-100 a 2,5% e subsequentemente lavado com 50 mM de Tris, pH 8,4. O gel foi subsequentemente incubado a 37°C durante a noite em uma solução de ativação contendo 50 mM de Tris, pH 8,4, 5 mM de CaCl2 e ZnCl2. A coloração foi realizada durante 2 horas com azul de Coomassie a 0,5%, e a descoloração foi realizada com metanol a 30% e ácido acético a 10% até que bandas claras fossem observadas sobre um fundo escuro. Os géis foram fotografados, e a intensidade da ação gelatinolítica (bandas claras) foi analisada em UVP, UV e um analisador de imagem White Darkhon.

### Análise estatística

Antes da análise estatística, todos os dados foram testados quanto à normalidade usando o teste Shapiro-Wilk. Os resultados foram expressos como média ± desvio padrão e submetidos ao teste t de Student para amostras independentes. O teste de correlação de Pearson foi utilizado para avaliar a associação entre as variáveis colágeno I, MMP-2, TIMP e leptina. Todas as análises estatísticas foram realizadas usando SigmaStat para Windows (Versão 3,5). O nível de significância considerado foi de 5 % (α = 0,05).

## Resultados

### Características gerais dos animais

As características gerais dos animais encontram-se na Tabela 1 . O peso corporal final; os depósitos de gordura epididimal, retroperitoneal e visceral; a GC total; e o IA foram significativamente mais altos no grupo obeso do que no grupo controle. Durante o período experimental, os animais no grupo obeso consumiram menos comida e calorias do que os animais do grupo controle; no entanto, a eficiência alimentar foi maior nos animais obesos.

**Tabela 1:** – Características gerais dos animais

Variáveis	Grupos		

	Controle (n = 20)	Obeso (n = 21)	Valor de p
PCI (g)	151 ± 11	151 ± 11	0,290
PCF (g)	480 ± 51	534 ± 58	0,009
Gordura epididimal (g)	9,3 ± 2,3	14,2 ± 3,4	< 0,001
Gordura epididimal/100g PCF	1,9 ± 0,5	2,7 ± 0,6	< 0,001
Gordura retroperitoneal (g)	10,5 ± 3,3	21,7 ± 5,9	< 0,001
Gordura retroperitoneal/100g PCF	2,2 ± 0,7	4,1 ± 1,1	< 0,001
Gordura visceral (g)	6,3 ± 1,4	11,2 ± 4,2	< 0,001
Gordura visceral/100g PCF	1,3 ± 0,3	2,1 ± 0,8	< 0,001
GC (g)	26,1 ± 6,2	47,2 ± 12,3	< 0,001
Índice de adiposidade	5,6 ± 0,9	8,8 ± 1,6	< 0,001
Consumo alimentar (g/dia)	22,8 ± 2,1	17,0 ± 2,3	< 0,001
Ingestão calórica (kcal)	67,4 ± 6,3	62,1 ± 8,2	0,03
Eficiência alimentar (%)	2,1 ± 0,2	2,7 ± 0,2	< 0,001

*Valores são média ± desvio padrão. GC: gordura corporal; PCF: peso corporal final; PCI: peso corporal inicial. Teste t de Student.*

### Perfis metabólicos e endócrinos

**Figura 1:**
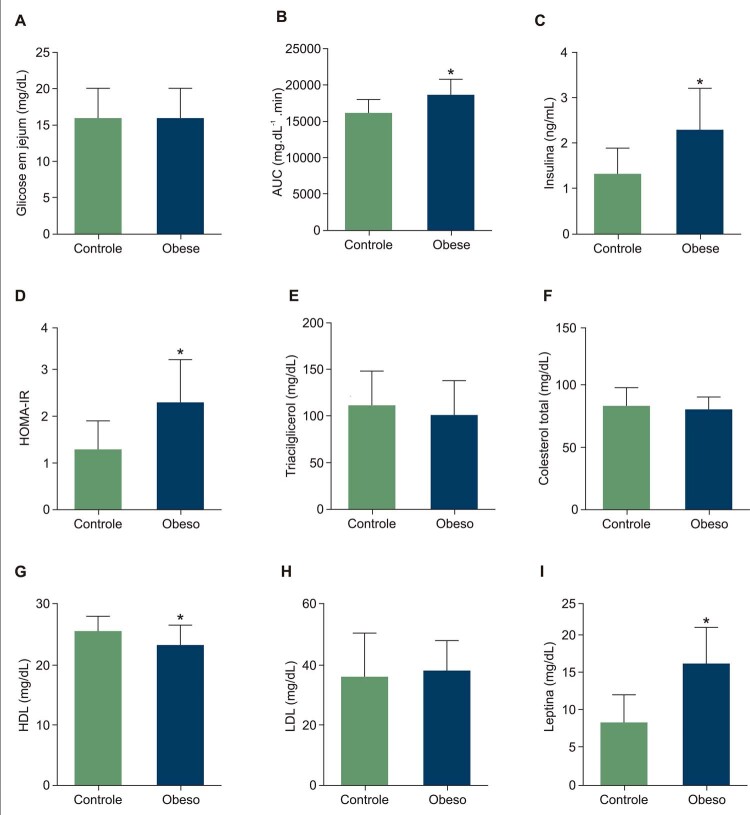
– Perfil metabólico e endócrino. (A) Glicose em jejum, (B) Área sob a curva (AUC) do teste intraperitoneal de tolerância à glicose, (C) insulina, (D) modelo de avaliação da homeostase da resistência à insulina (HOMA-IR), (E) triacilglicerol, (F) colesterol total, (G) lipoproteina de alta densidade (HDL), (H) lipoproteina de baixa densidade (LDL) e (I) leptina em ratos controles e obesos. (n = 15 – 21 por grupo). Dados são apresentados como média ± desvio padrão; teste t de Student. *: p < 0,05.

Os perfis metabólicos e endócrinos estão resumidos na Figura 1 . A obesidade a longo prazo induzida por alto teor de gordura levou a alterações metabólicas e hormonais significativas. Houve um aumento significativo na AUC de glicose, bem como nos níveis de insulina e leptina no grupo obeso, em comparação com o controle. Os animais obesos apresentaram um aumento da AUC, insulina sérica e HOMA-IR. As medidas séricas de glicose, triacilglicerol, colesterol total, HDL e LDL foram semelhantes entre os grupos.

### Pressão arterial sistólica e perfil morfológico cardíaco

A Tabela 2 demonstra que a PAS foi mais alta nos animais obesos, e não houve diferenças significativas entre os grupos para quaisquer parâmetros estudados em relação ao perfil morfológico, exceto no ventrículo direito, sugerindo que a obesidade não desencadeou hipertrofia do ventrículo esquerdo.

**Tabela 2:** – Pressão arterial sistólica e perfil morfológico cardíaco

Variáveis	Grupos		

	Controle (n = 20)	Obeso (n = 21)	Valor de p
PAS	127 ± 11,0	134 ± 12,0	0,04
Coração (g)	1,10 ± 0,10	1,17 ± 0,13	0,06
PA (g)	0,093 ± 0,018	0,094 ± 0,021	0,80
PVE (g)	0,81 ± 0,09	0,82 ± 0,10	0,62
PVD (g)	0,22 ± 0,03	0,24 ± 0,03	0,04
PA/PCF. 10^-3^	0,20 ± 0,03	0,18 ± 0,03	0,14
PVE /PCF. 10^-3^	1,72 ± 0,11	1,71 ± 0,12	0,44
PVD/PCF. 10^-3^	0,48 ± 0,09	0,47 ± 0,05	0,64

*Valores são médias ± desvio padrão. PA: peso atrial; PAS: pressão arterial sistólica; PCF: peso corporal final; PVD: peso do ventrículo direito; PVE: peso do ventrículo esquerdo; relações PA/PCF; PVE/PCF; PVD/PCF; 10^-3^ = 0,001. Teste t de Student.*

### Níveis proteicos miocárdicos de colágeno tipo I, TIMP-1, TIMP-2 e leptina

**Figura 2:**
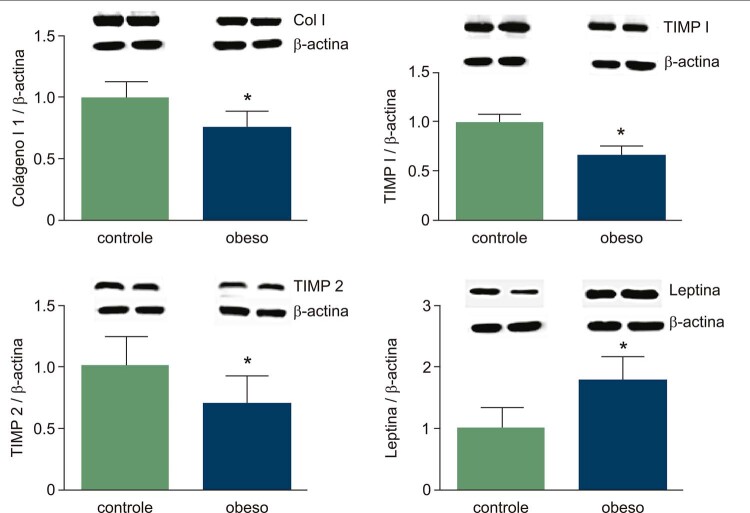
– Western blots representativos e quantificação de colágeno miocárdico tipo I, TIMP-1, TIMP-2 e leptina de ratos controles e obesos (n = 6 por grupo). As bandas dos western blot foram normalizados para β-actina. Dados são apresentados como média ± desvio padrão; teste t de Student. *: p < 0,05.

A Figura 2 demonstra que a obesidade promoveu uma redução dos níveis proteicos do colágeno tipo I, TIMP-1 e TIMP-2; no entanto, houve um aumento dos níveis proteicos de leptina no grupo obeso em comparação ao grupo controle.

### Atividade de MMP-2 miocárdica

**Figura 3:**
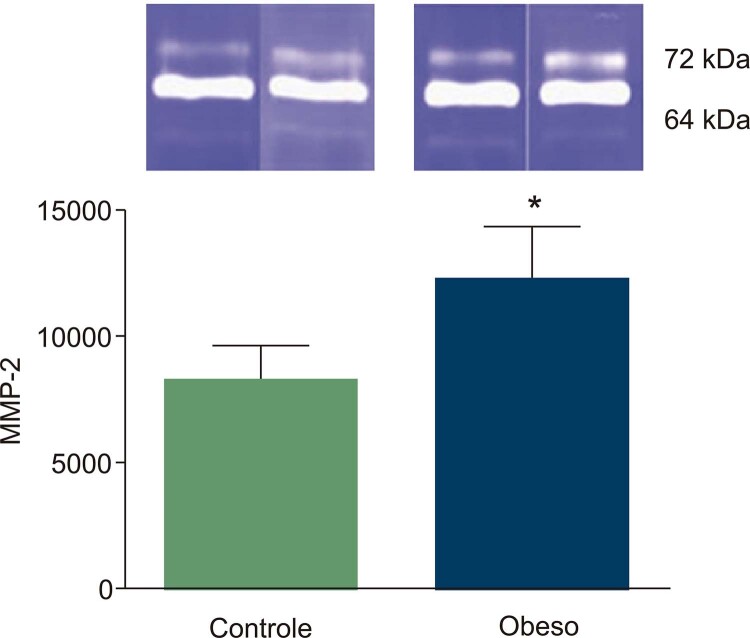
– Relação entre MMP-2 ativa (ativa e ativa intermediária) e inativa em ratos controles e obesos (n = 6 por grupo). MMP-2 inativa = 72 kDa e MMP-2 ativa = 64 kDa. Dados são apresentados como média ± desvio padrão; teste t de Student. *: p < 0,05.

A Figura 3 mostra a identificação de duas bandas fracas de degradação correspondentes à MMP-2 no gel de eletroforese: a MMP-2 inativa (pró-MMP-2) com um peso molecular de aproximadamente 72 kDa e MMP-2 ativa com um peso molecular de aproximadamente 64 kDa. Entre as duas bandas mencionadas, foi possível identificar a banda forte da degradação intermediária da MMP-2. Houve um aumento significativo na MMP-2 nos animais obesos.

### Associação linear entre variáveis cardíacas

A Tabela 3 demonstra que houve uma correlação significativa entre a redução do colágeno tipo I e o aumento da atividade da MMP-2, bem como entre a elevação da atividade da MMP-2 e a leptina. Além disso, foi observada uma correlação entre o aumento da MMP-2 e a redução do TIMP-1 e -2, bem como entre a redução do TIMP-1 e o aumento dos níveis proteicos de leptina.

**Tabela 3:** – Associação linear entre variáveis cardíacas

Associação	Coeficiente de correlação	Valor de p
Colágeno I × MMP-2	−0,723	0,008
MMP-2 × Leptina	0,766	0,004
MMP-2 × TIMP-1	−0,815	0,001
MMP-2 × TIMP-2	−0,597	0,040
TIMP-1 × Leptina	−0,656	0,020
TIMP-2 × Leptina	−0,273	0,390

*Valores são médias ± desvio padrão. PA: peso atrial; PAS: pressão arterial sistólica; PCF: peso corporal final; PVD: peso do ventrículo direito; PVE: peso do ventrículo esquerdo; relações PA/PCF; PVE/PCF; PVD/PCF; 10^-3^ = 0,001. Teste t de Student.*

## Discussão

O presente estudo visou investigar se a redução do colágeno tipo I está associada ao aumento da atividade da MMP-2 e a níveis elevados de leptina no miocárdio de ratos obesos. Os resultados principais confirmaram esta hipótese.

A alimentação contínua com uma dieta com alto teor de gordura foi eficaz para promover a obesidade em 34 semanas, uma vez que os ratos apresentaram níveis mais elevados de peso corporal, gordura e IA em comparação aos ratos alimentados com uma dieta padrão; tais dados corroboram outros estudos.^27 , 28^

As principais causas da obesidade são: o suprimento mais abundante de alimentos, a maior ingestão de alimentos palatáveis e densos em energia e a redução do gasto energético. A dieta com alto teor de gordura utilizada no presente estudo era rica em ácidos graxos mono e poliinsaturados com um conteúdo energético de 3,65 kcal/g, enquanto a dieta padrão dada ao grupo controle consistia em 2,95 kcal/g, gerando uma diferença de 24% em conteúdo calórico. Outros autores têm demonstrado que o consumo de uma dieta com alto teor de gordura promove menos saciedade e assim aumenta a ingestão de alimentos.^29^

Esses dados diferem dos nossos resultados, visto que os animais obesos ingeriram uma quantidade menor de alimentos e calorias em comparação ao controle. No entanto, a eficiência alimentar era mais alta nos ratos obesos, provavelmente devido ao efeito térmico do alimento. A gordura alimentar requer menos energia (2% – 3%) para ser metabolizada, e a gordura excessiva é, portanto, facilmente depositada na forma de triglicerídeos nos adipócitos, resultando em obesidade.^30^

Vários estudos têm relatado algumas comorbidades relacionadas à obesidade^29 , 31 , 32^ tais como intolerância à glicose, resistência à insulina, dislipidemia, hiperinsulinemia, hiperleptinemia e hipertensão arterial. No presente estudo, os animais obesos apresentaram maior AUC no teste oral de tolerância à glicose e maiores níveis séricos de insulina que os controles, indicando que a obesidade promoveu intolerância à glicose e hiperinsulinemia. A intolerância à glicose, associada ao aumento da insulina sérica, indicou que os ratos obesos apresentaram resistência à ação da insulina. Estes resultados são ainda corroborados pelo aumento do HOMA-IR nos ratos obesos. Todos estes achados estão de acordo com relatos prévios que utilizaram ratos alimentados com uma dieta com alto teor de gordura insaturada.^13 , 27 , 28 , 33^ Diversos estudos têm mostrado que a obesidade induzida por uma dieta com alto teor de gordura leva à dislipidemia,^19 , 34 , 35^ devido a mudanças em triacilglicerol, LDL ou HDL. Em nosso estudo, foi observada a redução dos níveis séricos de HDL. A leptina é um hormônio produzido pelo tecido adiposo, que participa do balanço energético, por meio da regulação da ingestão de alimentos e da oxidação de lipídios,^36^ e da biologia dos colágenos.^5 , 14 - 16^

Em relação aos efeitos da obesidade no sistema cardiovascular, a obesidade não promoveu remodelação cardíaca do ventrículo esquerdo. No entanto, a PAS aumentou nos animais obesos. Visto que o controle da PAS envolve o sistema neuro-humoral, como o sistema nervoso simpático e o sistema renina-angiotensina-aldosterona, os quais são aumentados na obesidade,^37^ pode-se inferir que o sistema neuro-humoral é ativado em animais obesos. Este achado está em acordo com alguns pesquisadores anteriores que investigaram a PAS em animais obesos alimentados com uma dieta com alto teor de gordura^38^ e em desacordo com outros.^27^

O objetivo principal deste estudo foi investigar se o aumento da atividade da MMP-2 pela leptina é responsável pela redução do colágeno miocárdico tipo I em ratos obesos. Os resultados desta investigação indicaram que houve uma redução dos níveis proteicos do colágeno tipo I acompanhada por um aumento da atividade da MMP-2 e dos níveis proteicos da leptina e uma redução dos níveis proteicos de TIMP-1 e TIMP-2 no coração. Como previamente referido, poucos estudos têm avaliado o comportamento do colágeno tipo I no miocárdio de animais com obesidade induzida por uma dieta com alto teor de gordura; enquanto Carroll e Tyaggi^12^ e Martínez-Martínez^39^ verificaram um aumento, Silva et al.,^13^ verificou uma redução do colágeno miocárdico tipo I.

As alterações no colágeno miocárdico podem resultar de uma elevação da síntese ou uma diminuição da degradação. Os dados deste estudo demonstraram que a degradação do colágeno tipo I pode ter prevalecido em ratos obesos, considerando que houve uma associação significativa entre o colágeno tipo I reduzido e o aumento da atividade da MMP-2. Embora alguns estudos indiquem que o aumento da atividade da MMP-2 melhora a síntese de colágeno,^40^ a maioria das informações na literatura indica o comportamento oposto, i.e., o aumento da atividade da MMP-2 promove a degradação do colágeno tipo I.^5 , 39 , 41^ Embora Martínez-Martínez et al.,^39^ e Zibadi et al.,^14^ tenham verificado que a leptina reduziu a atividade da MMP-2 *in vitro* , outros estudos têm demonstrado que a leptina aumenta a atividade da MMP-2 ^5 , 15 , 16^ e os nossos resultados corroboram ainda mais este último achado. Portanto, a elevação da MMP-2 pode ter sido consequente ao aumento da leptina cardíaca, porque houve uma estreita associação entre estas duas variáveis, embora esses achados não necessariamente reflitam uma relação de causa e efeito. Apesar disso, vários estudos têm relatado uma relação direta entre a leptina e a atividade da MMP-2 em cardiomiócitos.^5 , 15 , 16^

Apesar do fato que o aumento da atividade da MMP está associado à elevação da leptina cardíaca, outro fator modificante dessa enzima é o comportamento dos TIMP. Os resultados do presente estudo demonstraram uma redução dos níveis proteicos de TIMP-1 e TIMP-2 em animais obesos, que pode ter influenciado o aumento da MMP-2, uma vez que houve uma associação significativa entre MMP-2 e TIMP-1 e TIMP-2. A redução do TIMP-1 pode estar relacionada ao aumento da leptina, uma vez que houve uma associação significativa entre estas variáveis. Este achado é consistente com Schram et al., que verificaram uma redução substancial na expressão do mRNA de TIMP-1 após a elevação das concentrações da leptina em células cardíacas cultivadas.^15^ Até onde sabemos, este é o primeiro estudo que avalia a associação entre colágeno tipo I, leptina, MMP-2 e TIMP-1 e TIMP-2 no miocárdio de animais obesos alimentados com uma dieta com alto teor de gordura insaturada. No entanto, outras análises são necessárias para confirmar a relação de causa e efeito.

## Conclusão

Os achados confirmaram a hipótese de que a redução do colágeno tipo I está associada ao aumento da atividade da MMP-2, que está, por sua vez, ligada à elevação da leptina no miocárdio de ratos obesos. Este estudo permitiu a avaliação de mediadores envolvidos na remodelação cardíaca, os quais podem desencadear a função cardíaca comprometida na obesidade. A identificação desses fatores deletérios pode facilitar possíveis alvos terapêuticos.
