# Supplementary material for: A Redução do Colágeno Tipo I está Associada ao Aumento da Atividade da Metaloproteinase-2 e da Expressão Proteica de Leptina no Miocárdio de Ratos Obesos
Source: Arq Bras Cardiol. 2020 Jul 28;115(1):61–70. [Article in Portuguese] doi: 10.36660/abc.20180143 (PMC8384327; doi:10.36660/abc.20180143)
Supplement: Translation

# Translation: Decreased Collagen Type I is Associated with Increased Metalloproteinase-2 Activity and Protein Expression of Leptin in the Myocardium of Obese Rats

**Authors:** Danielle Cristina Tomaz da Silva-Bertani, Danielle Fernandes Vileigas, Gustavo Augusto Ferreira Mota, Sérgio Luiz Borges de Souza, Loreta Casquel De Tomasi, Dijon Henrique Salomé de Campos, Adriana Fernandes de Deus, Paula Paccielli Freire, Carlos Augusto Barnabe Alves, Carlos Roberto Padovani, Antonio Carlos Cicogna

**Affiliations:** 1 Brazil Faculdade de Medicina de Botucatu - Universidade Estadual Paulista (UNESP), Botucatu, SP - Brazil; 2 Brazil Instituto de Biociências - Universidade Estadual Paulista (UNESP), Botucatu, SP - Brazil

**Keywords:** Cardiovascular Diseases/physiopathology, Obesity, Collagen Type 1, Rats, Leptin, Adiposity, Tissue Inhibitor of Metalloproteinases, Metaloproteinase-2

## Abstract

**Background:**

Obesity is a risk factor for medical complications, including the cardiovascular system. There is limited information on collagen in the heart in obesity. Our previous study showed decreased protein levels of myocardial collagen type I in obese rats fed a high-fat diet for 34 weeks. However, the mechanisms responsible for low levels are not fully elucidated.

**Objective:**

The purpose of this study was to test the hypothesis that the reduction in collagen type I is associated with increased metalloproteinase-2 (MMP-2) activity, which is linked to elevated leptin in the myocardium of obese rats.

**Methods:**

Thirty-day-old male Wistar rats were randomized into two groups, control (standard diet) and obese (high-fat diet), and fed for 34 weeks. The general animal characteristics and metabolic and endocrine profiles were evaluated. Myocardial protein expressions of collagen I, leptin, tissue inhibitors of metalloproteinases (TIMP), and MMP-2 activity were assessed. Pearson correlation was employed to determine the associations between variables. The level of significance was 5%.

**Results:**

The obese animals had increased adiposity index compared to control. Comorbidities such as glucose intolerance, hyperinsulinemia, insulin resistance, hyperleptinemia, and hypertension were observed in obese rats. Obesity reduced collagen I, TIMP-1, and TIMP-2, and it increased leptin and MMP-2 in the myocardium. There was a negative correlation between collagen I and MMP-2 and a positive correlation between leptin and MMP-2.

**Conclusion:**

The hypothesis was confirmed; the reduction in collagen type I is associated with increased MMP-2 activity and leptin expression in the myocardium of obese rats. (Arq Bras Cardiol. 2020; 115(1):61-70)

## Introduction

Obesity is a chronic metabolic disorder characterized by excessive accumulation of adipose tissue. The prevalence of obesity has increased worldwide, representing a major public health problem that affects both developed and developing countries.^1 , 2^

The adipocytes are influenced by several substances, and they secrete numerous peptides that act directly or indirectly on the cardiovascular system. Therefore, adipose tissue is not simply an energy deposit, but also an active endocrine, paracrine and autocrine organ with multiple functions, including the ability to synthesize and release mediators, like leptin, that participate in multiple biological processes, including those that occur in the heart.^3^

The heart is composed of myocytes, nerves, vessels, and the extracellular matrix (ECM). The main component of the ECM is collagen, predominantly type I and III, with type I being the most abundant, corresponding to approximately 80% of total myocardial collagen.^4^ This protein is produced by fibroblasts and degraded by the family of matrix metalloproteinases (MMP).^5^ In a stable condition, it contributes to the maintenance of cardiac architecture and function.^6^ Several mechanisms act to ensure that the components of matrix degradation by MMP are precisely controlled, including tissue inhibitors of metalloproteinases (TIMP).^7^ Cardiac collagen changes in response to neuro-hormonal and mechanical stimuli,^6 , 8^ due to elevated synthesis and decreased degradation or vice versa.

Several studies have analyzed the expression of collagen type I in different tissues in experimental models of obesity.^9 - 11^ There is limited information on the behavior of this type of collagen in the heart in obese animals. Although Carroll et al.^12^ have shown elevated myocardial collagen type I in obese rabbits fed a high-fat diet for 12 weeks, a previous study by our group, Silva et al.,^13^ found decreased cardiac collagen type I in obese rats fed an unsaturated high-fat diet for 30 weeks.^13^ The mechanisms responsible for the decreased myocardial collagen type I, however, were not studied.

One of the possible mechanisms involved in myocardial type I collagen regulation is the increased leptin hormone.^5 , 14 - 16^ Supporting this hypothesis, most studies *in vitro* have shown that leptin increases MMP-2 activity,^5 , 15 , 16^ which is involved in the degradation of collagen type I. Therefore, the purpose of this study was to test the hypothesis that the reduction in myocardial collagen type I, associated with increased activity of MMP-2, is linked to elevation of leptin in obese rats.

## Materials and methods

### Animals and Experimental Protocol

After one week for acclimatization, 30-day-old male Wistar rats were randomly assigned, by lottery, to one of two groups: control (n = 20) and obese (n = 21). The sample size used in this study was based on the literature and on our previous studies.^13 , 17 - 19^ The control group was fed standard rat chow (RC Focus 1765, Agroceres^®^, Rio Claro, SP, Brazil) containing 12.3% of kilocalories from fat, 57.9% from carbohydrates, and 29.8% from protein, whereas the obese group was fed one of four alternating high-fat diets (RC Focus 2413, 2414, 2415, and 2416, Agroceres^®^, Rio Claro, SP, Brazil) containing 49.2% of kilocalories from fat, 28.9% from carbohydrates, and 21.9% from protein. The four high-fat diets had the same nutritional composition, except flavoring additives, namely, cheese, bacon, chocolate, or vanilla. Each diet was changed daily, and the rats were maintained on their respective diets for 34 consecutive weeks. The high-fat diet was calorically rich compared to the standard diet (3.65 kcal/g vs. 2.95 kcal/g) due to the higher fat composition. The high-fat diet consisted of saturated and unsaturated fatty acids, which provided 20% and 80% of the fat-derived calories, respectively.

Rats were housed in individual cages in an environmentally controlled clean-air room at 23 (± 3)ºC with a 12-hour light/dark cycle and 60% (± 5%) relative humidity. All experiments and procedures were conducted according to the Guide for the Care and Use of Laboratory Animals, published by the National Research Council (1996),^20^ and they received approval from the Botucatu Medical School Ethics Committee (UNESP, Botucatu, SP, Brazil, Protocol: 861-2011).

### Animal general characteristics and metabolic and endocrine profiles

Animal general characteristics and metabolic and endocrine profiles were evaluated according to the following parameters: body weight, body fat (BF), adiposity index (AI), food consumption, caloric intake, feed efficiency, glucose tolerance, insulin resistance, serum lipid profile, and serum leptin and insulin concentrations. A criterion based on the AI was used to determine obesity. The AI is an easy and consistent method used by several authors to evaluate the amount of BF in rodents.^21 - 23^

The animals’ food intake and body weight were measured weekly. Caloric intake was determined by multiplying the energy value of each diet (g × kcal) and weekly food intake. To analyze the animals’ capacity to convert consumed food energy to body weight, feed efficiency was calculated, dividing the total body weight gain (g) by total energy intake (Kcal).

Glucose tolerance was evaluated by the oral glucose tolerance test one week before euthanasia. After a 6-hour fast, blood samples were collected by puncture from the tail tip at baseline and after intraperitoneal administration of 30% glucose solution (Sigma-Aldrich®, St Louis, MO, USA), equivalent to 2.0 g/kg body weight. Blood glucose concentrations were analyzed at 0 minutes (baseline) and after 15, 30, 60, 90, and 120 minutes of glucose infusion, using a handheld glucometer (Accu-chek Advantage; Roche Diagnostics Co., Indianapolis, IN, USA). Glucose intolerance was assessed by the area under the curve (AUC) for glucose.

At the end of the experimental protocol, after fasting for 12 hours, animals were anesthetized (sodium pentobarbital 50 mg/kg, intraperitoneal injection), decapitated, and thoracotomized; the adipose tissue fat pads were dissected and weighed. BF was calculated as the sum the weight of the individual fat pads as follows: BF = epididymal fat + retroperitoneal fat + visceral fat. AI was calculated by the following formula: AI = (BF/final body weight) × 100. Blood samples were collected in heparinized tubes, centrifuged at 3,000 × g for 10 minutes at 4°C, and stored at −80°C for later analysis. Triacylglycerol, total cholesterol, and high- (HDL) and low-density lipoprotein (LDL) concentrations were determined using specific kits (BIOCLIN®, Belo Horizonte, MG, Brazil). Hormone levels of leptin and insulin were determined by enzyme-linked immunosorbent assay (ELISA), using commercially available kits (EMD Millipore Corporation, Billerica, MA, USA).

The homeostatic model assessment of insulin resistance (HOMA-IR) was used as an insulin resistance index, calculated according to the formula: HOMA-IR = [fasting glucose (mmol/L) × fasting insulin (μU/mL)]/22.5.^24^

### Cardiovascular profile

The cardiovascular profile of the animals was also assessed according to the following parameters: systolic blood pressure (SBP); cardiac tissue morphology; myocardial protein expression of collagen type I, TIMP-1, TIMP-2, and leptin; and MMP-2 activity.

### Systolic blood pressure

At the end of the experiment, one week before euthanasia, SBP was measured in conscious rats using the non-invasive tail-cuff method with an electro-sphygmomanometer, Narco BioSystems^®^(International Biomedical, Austin, TX, USA).^25^ Arterial pulsations were recorded in a computerized data acquisition system (Biopac Systems Inc., CA, USA). The average of two readings was recorded for each measurement.

### Morphological study

The hearts were removed and dissected following euthanasia and thoracotomy. Atria, left and right ventricles weights, and their respective relations with final body weight were determined to evaluate the presence of cardiac remodeling (i.e., presence or absence of hypertrophy).

### Myocardial protein levels of collagen type I, TIMP-1, TIMP-2, and leptin

Left ventricular tissue was analyzed by Western Blot to quantify protein levels of collagen type I, TIMP-1, TIMP-2, and leptin. Six samples were used in each group to ensure that all samples were analyzed in the same electrophoresis run in order to avoid inter-gel variations. Briefly, frozen left ventricle samples were homogenized using a Polytron device (Ika Ultra TurraxTM T25 Basic, Wilmington, USA) in a lysis buffer containing 10 mM Tris, pH 7.4, 100 Mm NaCl, 1 mM EDTA, 1 Mm EGTA, 1% Triton X-100, 10% glycerol, 0.1% sodium dodecyl sulfate (SDS), 0.5% deoxycholate, and phosphatase and protease inhibitors (Sigma-Aldrich). The homogenate was centrifuged at 4ºC for 20 minutes at 12,000 rpm. The supernatant was collected, and total protein content was determined by the Bradford Method. Samples (50 µg) were subjected to SDS-polyacrylamide gel electrophoresis (SDS-PAGE) in polyacrylamide gels (6% or 10% depending on molecular protein weight). After electrophoresis, proteins were electro-transferred to nitrocellulose membrane (BioRad Biosciences; NJ, USA). The blotted membrane was then blocked (5% nonfat dry milk, 10 mmol/L Tris-HCl, pH 7.6, 150 mmol/L NaCl, and 0.1% Tween 20) for 2 hours at room temperature and incubated with specific antibodies overnight at 4ºC. Subsequently, the blotted membrane was incubated for 1.5 hours at room temperature with peroxidase-conjugated anti-rabbit or anti-mouse secondary antibody (1:10,000 dilution), and then incubated with enhanced chemiluminescence (Amersham Biosciences, NJ, USA) and detected by autoradiography. Quantification analysis of the blots was performed using Scion Image software (Scion, based on NIH Image). Mouse monoclonal antibodies to collagen type I (1:10,000), TIMP-2 (1:1,000), and leptin (1:1,000) and rabbit monoclonal antibodies to TIMP-1 (1:1,000) and β-actin (1:1,000) were obtained from Abcam (Cambridge, USA) and Cell Signaling (Danvers, USA), respectively. Targeted bands were normalized to the expression of cardiac β-actin.

### Myocardial metalloproteinase-2 activity

Myocardial MMP-2 activity was determined as reported by Tyagi et al.^26^ . Six samples were used in each group to ensure that all samples were analyzed in the same electrophoresis run in order to avoid inter-gel variations. In brief, left ventricular tissues were homogenized in a buffer containing the following: Tris 50 mM, pH 7.4, NaCl 0.2 M, Triton-X 0.1% and CaCl2 10 mM. The homogenate was centrifuged at 4ºC for 20 minutes at 12,000 rpm. The supernatant was collected, and total protein content was determined by the Bradford Method (Bradford 1976). Samples were diluted in application sample buffer consisting of 0.5 M Tris, pH 6.8, 100% glycerol, and 0.05% bromophenol blue. The samples were loaded into the wells of 8% SDS-polyacrylamide containing 1% gelatin. Electrophoresis was carried out in a Bio-Rad apparatus at 80 V for 2 hours. The gel was removed and washed two times with 2.5% Triton-X-100 and then washed with 50 mM Tris pH 8.4. The gel was then incubated at 37°C overnight in an activation solution consisting of 50 mM Tris, pH 8.4, 5 mM CaCl2 and ZnCl2. Staining was performed for 2 hours with 0.5% Coomassie blue, and destaining was performed in 30% methanol and 10% acetic acid until clear bands were observed over a dark background. The gels were photographed, and the intensity of gelatinolytic action (clear bands) was analyzed in UVP, UV, and a White Darkhon image analyzer.

### Statistical analysis

Prior to statistical analysis, all data were tested for normality using the Shapiro-Wilk test. Results were expressed as mean ± standard deviation and submitted to Student’s t-test for independent samples. Pearson correlation was used to evaluate the association between the variables collagen I, MMP-2, TIMP, and leptin. All statistical analyses were performed using SigmaStat for Windows (Version 3.5). The level of significance considered was 5 % (α = 0.05).

## Results

### Animal general characteristics

The general animal characteristics are displayed in Table 1 . Final body weight; deposits of epididymal, retroperitoneal, and visceral fat; total BF; and AI were significantly higher in the obese group than in the control group. During the experimental period, animals in the obese group ate less food and calories than those in the control group; however, the feed efficiency was higher in obese animals.

**Table 1:** – Animal general characteristics

Variables	Groups		

	Control (n = 20)	Obese (n = 21)	p value
IBW (g)	151 ± 11	151 ± 11	0.290
FBW (g)	480 ± 51	534 ± 58	0.009
Epididymal fat (g)	9.3 ± 2.3	14.2 ± 3.4	< 0.001
Epididymal fat/100gFBW	1.9 ± 0.5	2.7 ± 0.6	< 0.001
Retroperitoneal fat (g)	10.5 ± 3.3	21.7 ± 5.9	< 0.001
Retroperitoneal fat/100gFBW	2.2 ± 0.7	4.1 ± 1.1	< 0.001
Visceral fat (g)	6.3 ± 1.4	11.2 ± 4.2	< 0.001
Visceral fat/100gFBW	1.3 ± 0.3	2.1 ± 0.8	< 0.001
BF (g)	26.1 ± 6.2	47.2 ± 12.3	< 0.001
Adiposity index	5.6 ± 0.9	8.8 ± 1.6	< 0.001
Food consumption (g/day)	22.8 ± 2.1	17.0 ± 2.3	< 0.001
Caloric intake (kcal)	67.4 ± 6.3	62.1 ± 8.2	0.03
Feeding efficiency (%)	2.1 ± 0.2	2.7 ± 0.2	< 0.001

### Metabolic and endocrine profiles

The metabolic and endocrine profiles are summarized in Figure 1 . Long-term obesity induced by high fat led to significant metabolic and hormonal changes. There was a significant increase in the glucose AUC, as well as in insulin and leptin levels in the obese group, compared to control. The obese animals presented increased AUC, serum insulin, and HOMA-IR. The serum measurements of glucose, triacylglycerol, total cholesterol, HDL, and LDL were similar between groups.

**Figure 1:** – Metabolic and hormonal profile. (A) Fasting glucose, (B) Area under curve (AUC) of intraperitoneal glucose tolerance test, (C) insulin, (D) homeostasis model assessment of insulin resistance (HOMA-IR), (E) triacylglycerol, (F) total cholesterol, (G) high-density lipoprotein (HDL), (H) low-density lipoprotein (LDL) and (I) leptin in control and obese rats. (n= 15 – 21 per group). Data are presented as mean ± SD; Student’s t-test. *: p < 0.05.

### Systolic blood pressure and cardiac morphological profile

Table 2 shows that SBP was higher in the obese animals, and there were no significant differences between the groups for any of the studied parameters concerning morphological profile, except for the right ventricle, suggesting that obesity did not trigger left ventricular hypertrophy.

**Table 2:** Values are means ± SD. IBW: initial body weight; FBW: final body weight; BF: body fat. Student’s t-test. – Systolic blood pressure and cardiac morphological profile

Variables	Groups		

	Control (n = 20)	Obese (n = 21)	p value
SBP	127 ± 11.0	134 ± 12.0	0.04
Heart (g)	1.10 ± 0.10	1.17 ± 0.13	0.06
ATW (g)	0.093 ± 0.018	0.094 ± 0.021	0.80
LVW (g)	0.81 ± 0.09	0.82 ± 0.10	0.62
RVW (g)	0.22 ± 0.03	0.24 ± 0.03	0.04
ATW/FBW.^10-3^	0.20 ± 0.03	0.18 ± 0.03	0.14
LVW/FBW.^10-3^	1.72 ± 0.11	1.71 ± 0.12	0.44
RVW/FBW.^10-3^	0.48 ± 0.09	0.47 ± 0.05	0.64

*Values are means ± SD. SBP: systolic blood pressure; LVW: left ventricle weight; RVW: right ventricle weight; ATW: atrial weight; FBW: final body weight; ATW/FBW; LVW/FBW; RVW/FBW ratio; 10^-3^= 0.001. Student’s t-test.*

### Myocardial protein levels of collagen type I, TIMP-1, TIMP-2, and leptin

Figure 2 reveals that obesity promoted a reduction in protein levels of collagen type I, TIMP-1 and TIMP-2; however, there was an increase in leptin protein levels in the obese group compared to the control group.

**Figure 2:** – Representative western blots and quantification of myocardial collagen type I, TIMP-1, TIMP-2, and leptin from control and obese rats (n = 6 per group). Western blot bands were normalized to β-actin. Data are presented as mean ± SD. Student’s t-test. *: p < 0.05.

### Myocardial MMP-2 activity

Figure 3 shows the identification of two weak bands of degradation corresponding to MMP-2 in electrophoresis gel: inactive MMP-2 (pro-MMP-2) with a molecular weight of approximately 72 kDa and active MMP-2 with a molecular weight of approximately 64 kDa. Between the two bands mentioned, it was possible to identify the strong band of MMP-2 intermediate degradation. There was a significant increase in MMP-2 in the obese animals.

**Figure 3:** – Relation between active (active and intermediate active) and inactive MMP-2 in control and obese rats (n = 6 per group). Inactive MMP-2 = 72 kDa and active MMP-2 = 64 kDa. Data expressed as mean ± standard deviation. Student’s t test. *: p < 0.05.

### Linear association between cardiac variables

Table 3 reveals that there was a significant correlation between the decrease in collagen type I and increased MMP-2 activity, as well as between the elevation of MMP-2 activity and leptin. Moreover, a correlation was observed between the increase in MMP-2 and the decrease in TIMP-1 and -2, as well as between decreased TIMP-1 and increased leptin protein levels. There was no significant correlation between decreased TIMP-2 and increased leptin.

**Table 3:** – Linear association between cardiac variables

Association	Coefficient of correlation	p value
Collagen I × MMP-2	−0.723	0.008
MMP-2 × Leptin	0.766	0.004
MMP-2 × TIMP-1	−0.815	0.001
MMP-2 × TIMP-2	−0.597	0.040
TIMP-1 × Leptin	−0.656	0.020
TIMP-2 × Leptin	−0.273	0.390

*MMP: metalloproteinase; TIMP: tissue inhibitor of metalloproteinases. Pearson’s correlation test. Control, n = 6 and obese, n = 6.*

## Discussion

This study aimed to investigate whether a reduction in collagen type I is associated with increased MMP-2 activity and elevated levels of leptin in the myocardium of obese rats. The main results confirmed this hypothesis.

The continuous feeding of a high-fat diet was effective to promote obesity in 34 weeks, given that the rats showed higher levels of body and fat weight and AI compared to rats fed a standard diet; these data are in agreement with other studies.^27 , 28^

The major causes of obesity are a more abundant supply of food, higher intake of energy-dense, palatable food, and reduction in energy expenditure. The high-fat diet used in the current study was rich in mono- and polyunsaturated fatty acids with an energy content of 3.65 kcal/g, while the standard diet fed to the control group consisted of 2.95 kcal/g, generating a difference of 24% in caloric content. Authors have shown that consumption of a high-fat diet promotes less satiety and thus increased food intake.^29^

These data differ from our results since the obese animals ate a lower amount of food and calories compared to control. However, feed efficiency was higher in obese rats, likely due to the thermic effect of food. Dietary fat requires less energy (2% – 3%) to be metabolized, and the excessive fat is thus easily deposited in the form of triglycerides in adipocytes, resulting in obesity.^30^

Several studies have reported some comorbidities related to experimental obesity,^29 , 31 , 32^ such as glucose intolerance, insulin resistance, dyslipidemia, hyperinsulinemia, hyperleptinemia, and arterial hypertension. In the present study, the obese animals exhibited higher AUC in the oral glucose tolerance test and higher serum insulin levels than controls, indicating that obesity promoted glucose intolerance and hyperinsulinemia. Glucose intolerance, associated with increased serum insulin, indicated that obese rats presented resistance to the action of insulin. These results are further supported by the increased HOMA-IR in obese rats. All these findings are in accordance with previous reports that used rats fed a high unsaturated fat diet.^13 , 27 , 28 , 33^ Different studies have shown that high-fat diet-induced obesity leads to dyslipidemia,^19 , 34 , 35^ whether due to changes in triacylglycerol, LDL, or HDL, in agreement with our study, which observed decreased serum HDL levels. Leptin is a hormone produced by adipose tissue, which participates in energy balance, by regulating food intake and the oxidation of lipids,^36^ and in the biology of collagens.^5 , 14 - 16^

Concerning the effects of obesity on the cardiovascular system, obesity did not promote left ventricular cardiac remodeling. However, SBP increased in obese animals. As SBP control involves the neurohumoral system, such as the sympathetic nervous system and the renin-angiotensin-aldosterone system, which are increased in obesity,^37^ it may be inferred that the neurohumoral system is activated in obese animals. This finding is consistent with some previous researchers who investigated SBP in obese animals fed a high-fat diet^38^ and in disagreement with others.^27^

The main objective of this study was to investigate whether increased MMP-2 activity by leptin is responsible for the reduction in myocardial collagen type I in obese rats. The results of this investigation indicated that there was a reduction of protein levels of collagen type I accompanied by an increase in MMP-2 activity and leptin protein levels and a decrease in TIMP-1 and TIMP-2 protein levels in the heart. As previously stated, few studies have evaluated the behavior of collagen type I in the myocardium of animals with obesity induced by a high-fat diet; while Carroll^12^ and Martínez-Martínez^39^ found an increase, Silva et al.^13^ found a decrease in myocardial type I collagen.

The changes in myocardial collagen may result from an elevation of the synthesis or decreased degradation. The data from this study showed that the degradation of collagen type I may have prevailed in obese rats, as there was a significant association between reduced collagen type I and increased MMP-2 activity. Although some studies show that the increase in MMP-2 activity enhances collagen synthesis,^40^ most information in the literature indicates the opposite behavior, i.e., the increase in MMP-2 activity promotes the degradation of collagen type I.^5 , 39 , 41^ Although Martínez-Martínez et al.^39^ and Zibadi et al.^14^ have found that leptin reduced the MMP-2 activity *in vitro* , other studies have shown that leptin increases MMP-2 activity,^5 , 15 , 16^ and our results further support this latest finding. Thus, the elevation of MMP-2 may have been consequent to increased cardiac leptin, because there was a close association between these two variables, although these findings do not necessarily reflect a cause-and-effect relationship. Nevertheless, several studies have reported a direct relation between leptin and MMP-2 activity in cardiomyocytes.^5 , 15 , 16^

Despite the fact that increased MMP activity is associated with elevated cardiac leptin, another modifying factor of this enzyme is the behavior of TIMP. The results of the present study showed a decrease in TIMP-1 and TIMP-2 protein levels in obese animals, which may have influenced the increase of MMP-2, given that there was a significant association between MMP-2 and TIMP-1 and TIMP-2. The reduced TIMP-1 may be related to the increase in leptin, as there was a significant association between these variables. This finding is consistent with Schram et al., who found a substantial reduction in TIMP-1 mRNA expression after the elevation of leptin concentrations in cultured cardiac cells.^15^ To our knowledge, this is the first study that evaluated the association between collagen type I, leptin, MMP-2, and TIMP-1 and TIMP-2 in the myocardium of obese animals fed an unsaturated high-fat diet. However, further analyses are required to confirm the cause-and-effect relationship.

## Conclusion

The findings confirmed the hypothesis that the reduction in collagen type I is associated with increased MMP-2 activity, which is in turn linked to an elevation of leptin in the myocardium of obese rats. This study allowed for evaluation of mediators involved in cardiac remodeling, which can trigger impaired heart function in obesity. The identification of these deleterious factors may enable possible therapeutic targets.
